# Supplementary material for: An exploration of the journey to diagnosis of Ehlers-Danlos Syndrome (EDS) for women living in Australia
Source: PLoS One. 2024 Jul 25;19(7):e0307574. doi: 10.1371/journal.pone.0307574 (PMC11271888; doi:10.1371/journal.pone.0307574)
Supplement: S1 Appendix — (DOCX) [file pone.0307574.s001.docx]

**Participant Survey:**

Prior to commencing this survey, can you please confirm the following:

- I have read the Participant Information Sheet: Version 1 and am satisfied with the information provided– tick box YES, NO.
- I identify as female (trans woman or non-binary person who was assigned female at birth) – tick box YES, NO.
- I am over 18 years old – tick box YES, NO.
- I was diagnosed with EDS in Australia – tick box YES, NO.
- I lived in Australia for at least one year prior to my EDS diagnosis – tick box YES, NO.

By clicking through, completing, and submitting this survey, I consent to participating in this study – tick box YES, NO.

Please complete the following 22 questions.

1. Age category:

- 18-24
- 25-34
- 35-44
- 45-54
- 55-64
- 65+
- Prefer not to say

1. Which level of Education did you complete?

- Finished high school before year 12
- finished high school (year 12)
- Completed TAFE/college studies
- Completed university studies (Bachelor’s/undergraduate degree)
- Completed post graduate studies
- Prefer not to say

1. Employment status (tick all that apply)

- Full time
- Part time
- Casual
- Homemaker
- Retired
- Studying
- Business owner
- Unemployed
- Receiving Disability Support Pension (DSP)
- Other (please specify)
- Prefer not to say

1. Which type of EDS do you have?

- Classical EDS
- Classical-like EDS
- Cardiac-valvular EDS (cvEDS)
- Vascular EDS (vEDS)
- Hypermobile EDS (hEDS)
- Arthrochalasia EDS (aEDS)
- Dermatosparaxis EDS (dEDS)
- Kyphoscoliotic EDS (kEDS)
- Brittle Cornea Syndrome (BCS)
- Spondylodysplastic EDS (spEDS)
- Musculocontractural EDS (mcEDS)
- Myopathic EDS (mEDS)
- Periodontal EDS (pEDS)
- Other (please specify)

1. Do you have a family member who has been diagnosed with EDS in Australia?

- Yes, if so, please state which family member/s have been diagnosed
- No

1. When did you obtain your diagnosis?

- 2020 – present
- 2010 – 2020
- 2000 – 2010
- Before 2000

1. How long prior to obtaining your official diagnosis had you noticed symptoms?

- Less than 1 year
- 1-3 years
- 3-5 years
- 5-10 years
- 10-15 years
- More than 15 years ago

1. Which medical professional/s did you attend prior to obtaining your diagnosis? You can select more than one health professional.

- GP
- Rheumatologist
- Immunologist
- Cardiologist
- Physiotherapist
- Other (please specify)

1. Were you misdiagnosed during your journey?

- Yes
- No – Skip to Question 13
- Unsure – Skip to Question 13
- Prefer not to say – Skip to Question 13

1. How many misdiagnoses did you endure?

- 1-3
- 4-6
- 7-9
- more than 9

1. If you feel comfortable to, please identify the misdiagnoses you received:

- Chronic Fatigue Syndrome
- Myalgic Encephalomyelitis (ME)
- Chronic pain
- Generalised joint laxity
- Anxiety and/or Depression
- Other Psychological
- Other Physical
- Marfan Syndrome
- Dysautonomia
- If other, please specify

1. How did you feel after being misdiagnosed?

Box provided to type response

1. At some point in your journey, did you seek self-diagnosis online?

- Yes
- No – please skip to Q 16.
- Other (please specify)

1. Was the information available online helpful?

- Yes
- No
- Somewhat
- Comment box ‘please add additional comments:’

1. If yes, which media sources were most helpful to you?

- Facebook support groups
- Websites
- Medical journal articles
- Podcasts
- YouTube lectures/videos

Box to type others

Additional comments

1. Which formal tests, if any, were used that led to your diagnosis of EDS? Please select all that apply.

- Beighton scale
- Clinical criteria
- International diagnostic checklist for hEDS
- Presence of other potentially associated disorders
- Self-report of pain/other symptoms
- Other (please specify)
- Don’t know

1. Did one (or more) health professional/s treat you well?

- Yes

If yes, what was it that made your experience positive? Box to type comments

- No

If not, what was it that made your experience negative? Box to type comments

1. Which medical professional ultimately correctly identified EDS?

- Rheumatologist
- Geneticist
- Other (please specify)

1. How did you feel *after* you received your diagnosis?

Box to type response

1. What do you think might improve the journey to diagnosis of EDS for other women living in Australia?

Box to type response

1. Do you feel like you have had to educate any medical professionals about EDS?

- Yes
- No
- If so, which one/s? box to type

1. How do you think information might be better distributed to treating health professionals or the broader community to increase awareness of EDS?

Box to type response

This survey is now complete.

Many thanks again for your time and energy to complete this survey. Your effort is greatly appreciated. As a gentle reminder, and to further protect your identity, please do not record ‘done’ on this Facebook page/elsewhere. Results will be collated, analysed, and published in due course. Thank you again for your assistance with this research.
