# Supplementary material for: An exploration of the journey to diagnosis of Ehlers-Danlos Syndrome (EDS) for women living in Australia
Source: PLoS One. 2024 Jul 25;19(7):e0307574. doi: 10.1371/journal.pone.0307574 (PMC11271888; doi:10.1371/journal.pone.0307574)
Supplement: Attachment — Submitted filename: Reviewer response PONE-D-24-18819.docx [file pone.0307574.s002.docx]

Reviewer response

The authors have presented a generally well-written manuscript about the often frustrating and convoluted diagnostic journey for Australian women with EDS. This topic is significant, because although EDS diagnosis delays have been described in other countries, this is the first time exploring the issue in Australia. The topic is in line with this journal’s scope. The grammar, spelling, language, and style are appropriate for the topic and journal. The paper is well-organized and coherent. I particularly appreciate the well-developed section on “Looking to the future,” since so many studies on EDS diagnosis delays treat that superficially.

I identify no fatal flaws. I have the following Issues and suggestions:

| Page | Comment | References (if applicable) |
| --- | --- | --- |
| 2 | Abstract/Results: “More than three quarters of respondents were misdiagnosed.” The issue is the term “misdiagnosed” which I discuss below. |  |
| 3 | Introduction: “Ehlers-Danlos syndrome (EDS) is a rare heritable connective tissue disorder. Note the term EDS is currently referred to in the plural, e.g. “Ehlers-Danlos syndromes (EDS)” to denote multiple types.  Also, recommend a different reference, rather than the EDS website. | Recommend reference to Kulas Søborg et al., 2017, rather than EDS website. |
| 12 | Paragraph 2. “The majority of participants (n = 129, 84.9%) were misdiagnosed before being correctly diagnosed with EDS.” I recommend you not refer to these conditions as misdiagnoses, but rather as “diagnoses before EDS”, “diagnoses prior to EDS that respondents believed to be misdiagnoses”, or “comorbid diagnoses.” Such diagnoses may be incorrect or may co-exist with EDS but not fully explain all symptoms. The complex interactions among comorbidities and EDS is still being explored, but to label all earlier diagnoses as “misdiagnoses” is misleading. It is, however, entirely appropriate to discuss respondents’ reactions to receiving an earlier diagnosis which was unexpected or perceived as incorrect. So, your findings are relevant and important. Just need to adjust the terminology. | (Halverson et al., 2021) “[Participants] received numerous comorbid diagnoses...many of which they believed were inaccurate” (p. 4)  (Castori, 2020)  Suggests the wide range of joint hypermobility-related comorbidities in children is more an indicator of severity than a diagnostic discriminator (p. 127) |
| 10 | Table 2: Types of EDS. The term “hEDS” was created in 2017 nosology, but several respondents were diagnosed before then. Did any respondent indicate a diagnosis of an earlier label for hEDS, such as EDS-hypermobility type (EDS-HT) or EDS-Type 3 (EDS-III)? If so, did the researchers include them in the hEDS category? |  |
| 9 | The following tables need to be reorganized to facilitate clarity/readability:  **List data in the following tables/sections in descending order based on n/percent:**  **Table 1: Sections:**   - State of residence - Educational level. Also, The frequency (n) column in this table adds up to 168, not the sample size of 152. Please correct or indicate if participants could select more than one.   **Table 2 – Types of EDS**   - Omit all types with a frequency of 0 - The frequency (n) column adds up to 153 rather than the sample size of 152. Please correct or indicate if participants could select more than one.   **Table 5: Sections:**   - Professional consulted before formal diagnosis - Misdiagnosis   **Table 8: Section:**   - Helpful websites   **Table 9: Sections:**   - Health Professional responsible for diagnosis of EDS - Diagnostic tools used   **Table 11: Sections:**   - If yes, which health professionals? |  |
| 25 | “Cultural healthcare factors and cultural bias, where participants or researchers hold  biased views due their cultural backgrounds, may limit the generalisability of these  results to the global population of females with a diagnosis of EDS.” Can you give a specific example how this might have applied to this particular study? If not, I would remove this overly general statement. |  |

I thank the authors and editor for the opportunity to review this manuscript. Overall, I believe this is a well-written paper on an important topic to the EDS community. I recommend revise and resubmit to address the issues noted above.

References:

Castori, M. (2020). Joint hypermobility in children: A neglected sign needing more attention. *Minerva Pediatrica, 72*(2), 123-133. <https://doi.org/10.23736/S0026-4946.20.05766-7>

Halverson, C. M. E., Clayton, E. W., Sierra, A. G., & Francomano, C. (2021). Patients with Ehlers-Danlos syndrome on the diagnostic odyssey: Rethinking complexity and difficulty as a hero’s journey. *American Journal of Medical Genetics, Part C: Seminars in Medical Genetics, 187*(4), 416-424. <https://doi.org/10.1002/ajmg.c.31935>

Kulas Søborg, M.-L., Leganger, J., Mortensen, L. Q., Rosenberg, J., & Burcharth, J..(2017). Establishment and baseline characteristics of a nationwide Danish cohort of patients with Ehlers-Danlos syndrome. *Rheumatology, 56*(5), 763-767. https://doi.org/10.1093/rheumatology/kew478
