# Supplementary material for: An exploration of the journey to diagnosis of Ehlers-Danlos Syndrome (EDS) for women living in Australia
Source: PLoS One. 2024 Jul 25;19(7):e0307574. doi: 10.1371/journal.pone.0307574 (PMC11271888; doi:10.1371/journal.pone.0307574)
Supplement: Attachment — Submitted filename: Response table.docx [file pone.0307574.s003.docx]

Response Tables

**Reviewer 1:**

| Page | Comment | Response |
| --- | --- | --- |
| 2 | Abstract/Results: “More than three quarters of respondents were misdiagnosed.” The issue is the term “misdiagnosed” which I discuss below. | Edited as recommended. |
| 3 | Introduction: “Ehlers-Danlos syndrome (EDS) is a rare heritable connective tissue disorder. Note the term EDS is currently referred to in the plural, e.g. “Ehlers-Danlos syndromes (EDS)” to denote multiple types.  Also, recommend a different reference, rather than the EDS website. | Thank you for the suggested reference. We decided to use Malfait et al. (2020) instead as it specifically references EDS as a plural and is already part of the reference list. |
| 12 | Paragraph 2. “The majority of participants (n = 129, 84.9%) were misdiagnosed before being correctly diagnosed with EDS.” I recommend you not refer to these conditions as misdiagnoses, but rather as “diagnoses before EDS”, “diagnoses prior to EDS that respondents believed to be misdiagnoses”, or “comorbid diagnoses.” Such diagnoses may be incorrect or may co-exist with EDS but not fully explain all symptoms. The complex interactions among comorbidities and EDS is still being explored, but to label all earlier diagnoses as “misdiagnoses” is misleading. It is, however, entirely appropriate to discuss respondents’ reactions to receiving an earlier diagnosis which was unexpected or perceived as incorrect. So, your findings are relevant and important. Just need to adjust the terminology. | Edited within the introduction and results sections as recommended. |
| 10 | Table 2: Types of EDS. The term “hEDS” was created in 2017 nosology, but several respondents were diagnosed before then. Did any respondent indicate a diagnosis of an earlier label for hEDS, such as EDS-hypermobility type (EDS-HT) or EDS-Type 3 (EDS-III)? If so, did the researchers include them in the hEDS category? | No – while there was a space for comments, none of the hEDs participants spoke of a changing diagnosis due to the 2017 nosology. |
| 9 | The following tables need to be reorganized to facilitate clarity/readability:  **List data in the following tables/sections in descending order based on n/percent:**  **Table 1: Sections:**   - State of residence - Educational level. Also, The frequency (n) column in this table adds up to 168, not the sample size of 152. Please correct or indicate if participants could select more than one.   **Table 2 – Types of EDS**   - Omit all types with a frequency of 0 - The frequency (n) column adds up to 153 rather than the sample size of 152. Please correct or indicate if participants could select more than one.   **Table 5: Sections:**   - Professional consulted before formal diagnosis - Misdiagnosis   **Table 8: Section:**   - Helpful websites   **Table 9: Sections:**   - Health Professional responsible for diagnosis of EDS - Diagnostic tools used   **Table 11: Sections:**   - If yes, which health professionals? | Table 1: Reordered as recommended and also added a note to indicate that some participants chose more than one option for educational level.  Table 2: All types with zero frequency have been omitted and numbers now add to 152  Table 5: Reordered as recommended  Table 8,9 and 11: Reordered as recommended |
| 25 | “Cultural healthcare factors and cultural bias, where participants or researchers hold  biased views due their cultural backgrounds, may limit the generalisability of these  results to the global population of females with a diagnosis of EDS.” Can you give a specific example how this might have applied to this particular study? If not, I would remove this overly general statement. | Removed as recommended. |

**Reviewer 2:**

Introduction:

| The study specifically addresses the prevalence and clinical characteristics of hypermobile Ehlers-Danlos Syndrome (hEDS). Given the distinct nature and higher prevalence of hEDS compared to other EDS subtypes, it is imperative to focus exclusively on hEDS to provide accurate and clinically relevant data.  Several statements in the article represent inaccurate information about hEDS, including:  o “EDS alters collagen structures in the body leading to multi-systemic complications including generalized joint hypermobility,…” and “functional decline is common place.” The etiology of hEDS is unknown, and it is generally not caused by defective collagen. The condition is not progressive. | The information regarding hEDS has been expanded to clearly differentiate it from other types of EDS and improve the clarity of this section. |
| --- | --- |
| Several reports indicate that over 70% of EDS cases occur in women with over 90% of hEDS cases being female.” While hEDS is more common in females, there is no sex predominance in other EDS subtypes. | This has been corrected as recommended. |
| Due to the ‘invisible’ nature of EDS, patient reports of their symptoms, particularly before they are accurately diagnosed with EDS, are often dismissed.” The term "invisible" is not appropriate; the authors likely refer to the lack of a pathognomonic biomarker. Additionally, this statement refer specifically to hEDS and should be clarified. | This has been corrected as recommended. |

Methods:

| The convenience sampling method used in the study is prone to biases that cannot be generalized beyond the specific sample studied. The authors should present their data as an exploratory or pilot study, generating hypotheses for more rigorous research designs using probability sampling methods. This approach would prevent the overstatement of findings and acknowledge the limitations inherent to convenience sampling | The methodology now contains a section which describes the study as a pilot study as recommended. |
| --- | --- |
| Authors grouped all Ehlers-Danlos syndrome (EDS) subtypes together, and provided a prevalence. The method used estimated the prevalence of EDS globally and applies it to the Australian population. Their statistical assessment based on a presumed and inaccurate prevalence, considering the variable and largely unknown prevalence of individual EDS subtypes.  . | The prevalence for EDS in a number of developed countries is approximately 120 per 100,000. As there is no data available specifically for Australia, this seems like the most reliable reference to use to determine a sample size.  The wording in this section has been simplified.  The authors welcome suggests to improve this section if still not deemed to be suitable. |

Results:

| Revision of all these statements are required:  “The majority of participants (n=129; 84.9%) were misdiagnosed before being correctly diagnosed with EDS. Most participants were diagnosed with anxiety and/or depression (n=91; 73.4%) or another psychological condition (n=47; 37.9%), chronic pain (n=67; 54%), myalgic encephalomyelitis/chronic fatigue syndrome (ME/CFS) (n=58; 46.8%), or another physical condition including fibromyalgia (n=18; 17.7%). Over 10% of respondents (n=14) received more than nine misdiagnoses before being correctly diagnosed with EDS.”  These statements are stemmed from a question that does not specify the timeframe, context, or criteria for what constitutes a "misdiagnosis (questions 11, and 16. This lack of clarity can lead to inconsistent interpretations and responses from participants. Without rigorous validation and a clear, objective framework for assessing misdiagnoses, self-reported data on this topic are likely to be inaccurate and unreliable, making all the conclusions unreliable. | These statements have been revised as recommended. |
| --- | --- |

Discussion:

| Revision of all these statements are required:  “Most participants were misdiagnosed prior to receiving their formal diagnosis of EDS with over ten percent of respondents receiving more than nine misdiagnoses.”  “Participants consistently reported being referred to numerous specialists who lack knowledge regarding EDS, often resulting in misdiagnosis.”  “While misdiagnosis is expected and consistent with previous findings the extent of misdiagnosis reported in this study is astonishing.”  These statements are stemmed from a question that does not specify the timeframe, context, or criteria for what constitutes a "misdiagnosis (questions 11, and 16. This lack of clarity can lead to inconsistent interpretations and responses from participants. Without rigorous validation and a clear, objective framework for assessing misdiagnoses, self-reported data on this topic are likely to be inaccurate and unreliable, making all the conclusions unreliable. | All of these statements have been revised as recommended. |
| --- | --- |
| The statement, "As FM alone is not formally recognized by the National Disability Insurance Agency in Australia (NDIA) or Centrelink (Australian welfare system), there is much debate about how helpful a diagnosis of FM is and whether FM is diagnosed in the absence of an alternative clear diagnosis," requires revision for several reasons:  Fibromyalgia (FM) is recognized globally and has been assigned an ICD code (International Classification of Diseases) long before hypermobile Ehlers-Danlos Syndrome (hEDS). The ICD-10 code for FM is M79.7, and it has been widely used in medical diagnosis and research. This recognition reflects the consensus within the medical community about FM as a legitimate and distinct medical condition.  The American College of Rheumatology has established specific diagnostic criteria that healthcare providers use to diagnose FM based on clinical findings. The lack of formal recognition by specific national agencies, such as the NDIA or Centrelink, does not diminish the validity of the diagnosis itself. It is important to distinguish between administrative recognition for the purposes of disability support and the medical validity of the diagnosis. The statement conflates these two distinct issues, potentially misleading readers about the legitimacy of FM as a clinical diagnosis.  In addition FM can co-occur with other conditions. Misrepresenting the status of FM can contribute to stigma and misunderstanding, which can negatively impact patients seeking support and treatment.  Therefore, it is recommended that the statement be revised to reflect the established medical recognition of fibromyalgia and clarify the distinction between administrative recognition for disability support and medical diagnosis. An accurate portrayal will ensure the article is informative and respectful of the condition's legitimacy. | Statements have been revised as recommended. |
| The statement, "While Physiotherapists made only 6.6% of the EDS diagnoses of participants in this study, this group of allied health professionals are aptly positioned to diagnose and treat EDS in conjunction with referrals to other appropriate specialists," requires clarification.  While physiotherapists play a crucial role in the multidisciplinary care of patients with hypermobile Ehlers-Danlos Syndrome (hEDS), their primary expertise lies in the management and rehabilitation of musculoskeletal issues, rather than in the diagnosis of complex genetic conditions. According to the 2017 International Classification of the Ehlers-Danlos Syndromes, the diagnosis of hEDS includes the exclusion of alternative diagnoses, which involves a comprehensive clinical assessment that includes detailed patient history, physical examination, and the exclusion of other conditions with overlapping phenotypes. This process typically requires the expertise of a medical professional trained in genetics, rheumatology, or another relevant specialty. Physiotherapists do not possess the necessary training or authority to perform the required comprehensive diagnostic workup. | This section has been revised as recommended. |

Limitations:

| The convenience sampling method used in the study is prone to biases that cannot be generalized beyond the specific sample studied. The authors should present their data as an exploratory or pilot study, generating hypotheses for more rigorous research designs using probability sampling methods. This approach would prevent the overstatement of findings and acknowledge the limitations inherent to convenience sampling. | This limitation has been added to the Limitations section. |
| --- | --- |
| The initial questionnaire design was based on an extensive literature search and the lead researcher’s anecdotal experience as an occupational therapist. While practical insights are valuable, the lead researcher’s expertise is in managing EDS complications rather than diagnosing it. Including an expert in EDS diagnosis, such as a geneticist or rheumatologist, would’ve ensured the questionnaire's accuracy and relevance. The authors should detail the specific expertise of the second researcher, clearly outlining their qualifications and experience in relation to EDS. | This limitation has been added to the Limitations section. |
| Patients' perceptions of being misdiagnosed, and their understanding of what diagnostic tool has been used, can be highly subjective and influenced by their understanding of their symptoms and conditions. The question does not include a mechanism for verifying the reported misdiagnoses against medical records or clinical evaluations. Without validation from healthcare professionals, it is impossible to determine if the reported misdiagnoses are accurate. | This limitation has been added to the Limitations section. |
| The authors should explicitly include self-selection bias, inaccurate or false data, and recall bias as limitations of the study. Recognizing these biases will provide a more balanced interpretation of the study’s findings. | These limitations have been added to the Limitations section. |
